# Supplementary material for: Direct spinning and densification method for high-performance carbon nanotube fibers
Source: Nat Commun. 2019 Jul 4;10:2962. doi: 10.1038/s41467-019-10998-0 (PMC6609687; doi:10.1038/s41467-019-10998-0)
Supplement: Supplementary file 1 — Supplementary Information [file 41467_2019_10998_MOESM1_ESM.pdf]

Supplementary Information

Direct spinning and densification method for high-performance carbon nanotube fibers

Lee et al.

Supplementary Figures

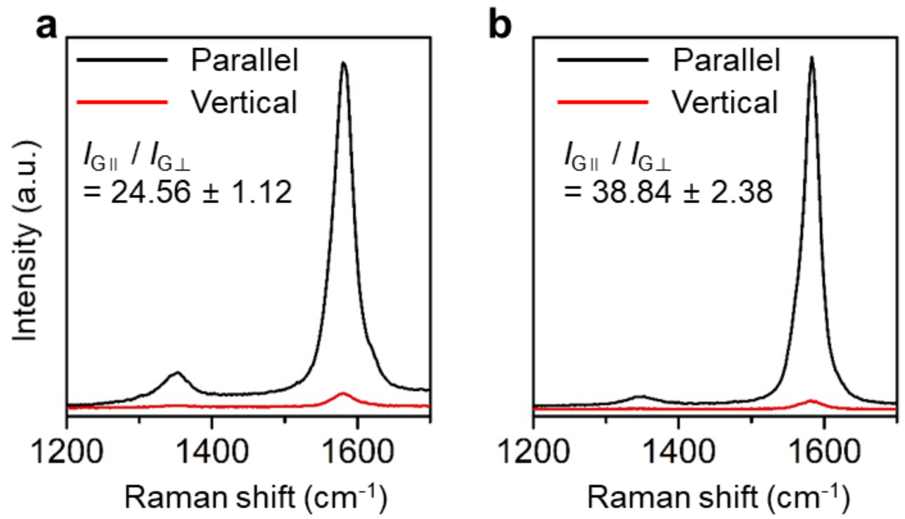

Supplementary Figure 1. Alignment of as-spun CNTFs. Polarized Raman spectra of as-spun CNTFs with different spinning rate. (a) 5 m min^{-1} and (b) 9 m min^{-1}.

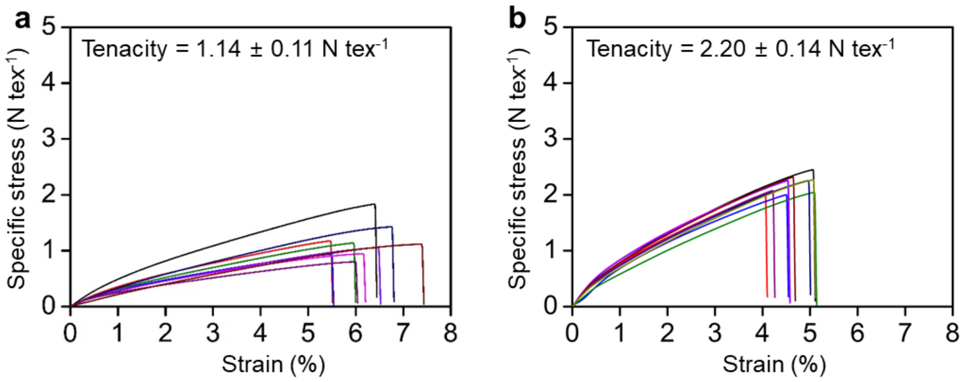

Supplementary Figure 2. Strength of as-spun CNTFs. Specific stress-strain curves of as-spun CNTFs with different spinning rate. (a) 5 m min^{-1} and (b) 9 m min^{-1}.

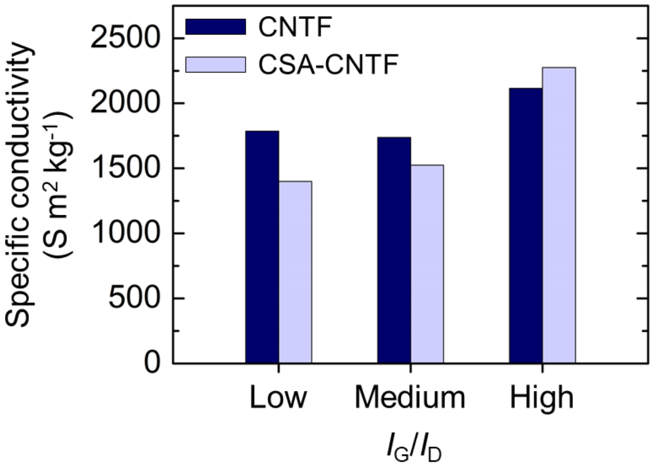

Supplementary Figure 3. Electrical conductivity of CNTFs. Specific electrical conductivity of CNTFs having low, medium, and high I_G/I_D before and after CSA treatment.

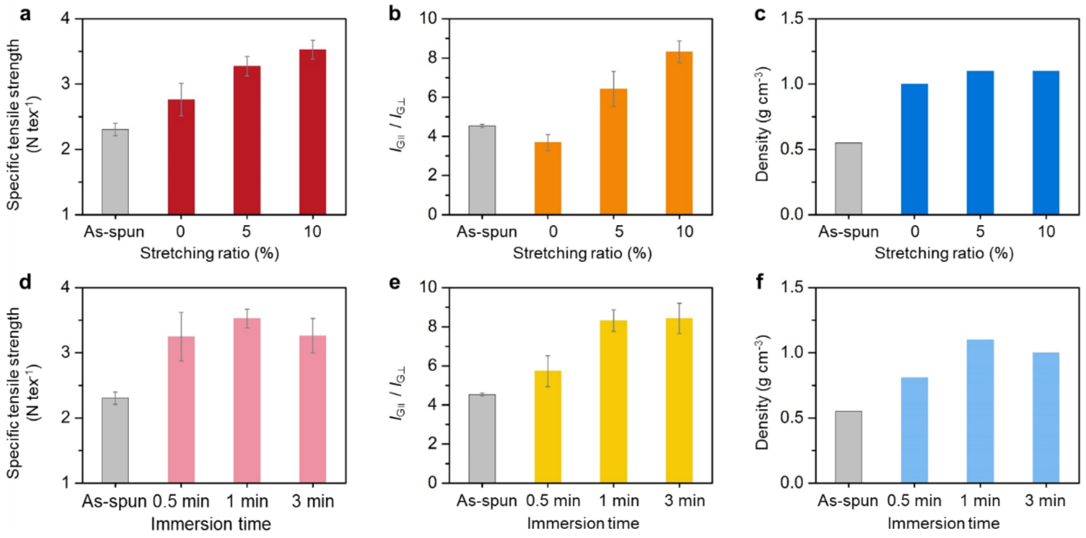

Supplementary Figure 4. Optimization of CSA densification process. Evolutions in (a) specific tensile strength, (b) $I_{G\parallel}/I_{G\perp}$, and (c) density of CNTFs having the medium I_G/I_D during CSA densification process with various stretching ratios (immersion time, 1 min). Evolutions in (d) specific tensile strength, (e) alignment, and (f) density of CNTFs with various immersion times (stretching ratio 10%). These are the real values of the data shown in Fig. 3. Error bars represent the standard deviation. Number of replicates for each experimental condition is as follows; (a) as-spun (n = 4), 0% (n = 5), 5% (n = 5), and 10% (n = 8), (b) n = 5 for all cases, (d) as-spun (n = 4), 0.5 min (n = 6), 1 min (n = 8), and 3 min (n = 10), and (e) n = 5 for all cases.

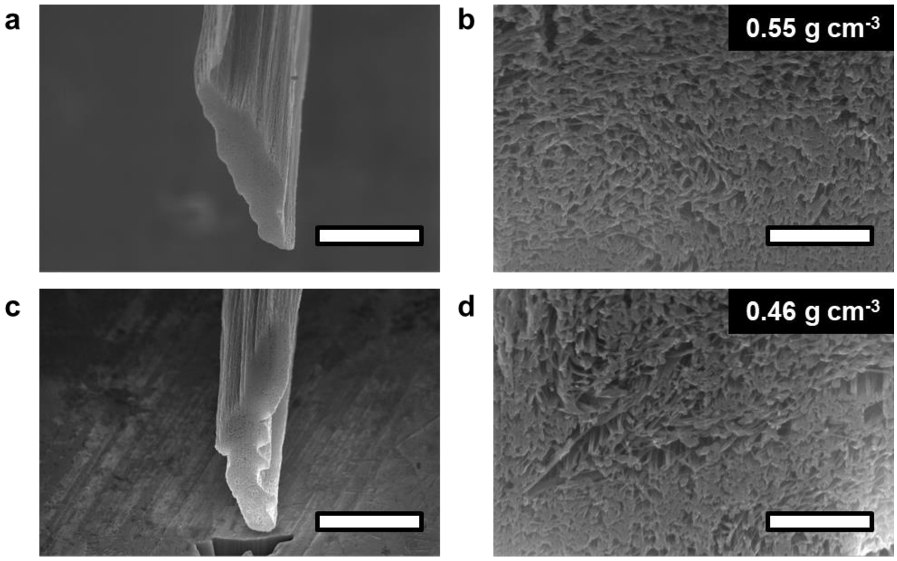

Supplementary Figure 5. Effect of acetone treatment. Cross-sectional SEM images of CNTFs (a)-(b) before and (c)-(d) after immersion in acetone for 1 min and drying in air. The SEM images in (b) and (d) are the magnified images of CNTFs in (a) and (c), respectively. The scale bars in (a) and (c) are 5 μm and those in (b) and (d) are 1 μm.

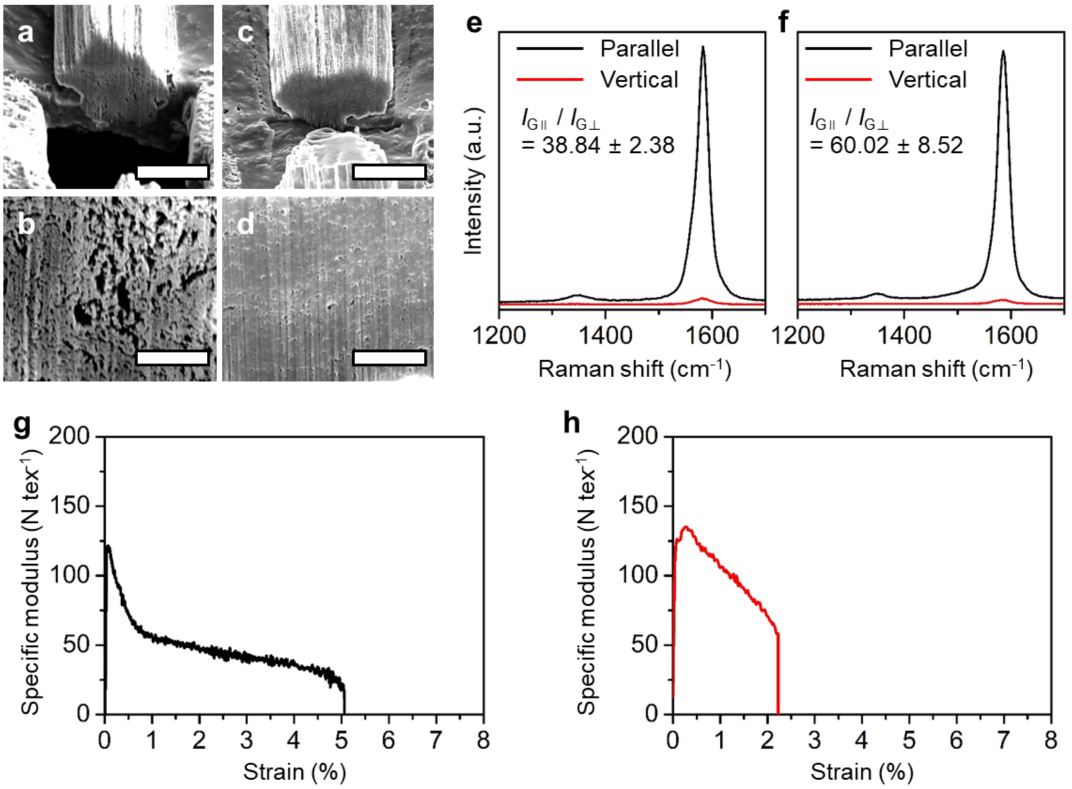

Supplementary Figure 6. Structural change of CNTFs with low I_G/I_D after CSA treatment. Cross-sectional SEM images of (a)-(b) as-spun CNTF and (c)-(d) CSA-CNTF. Polarized Raman spectra of (e) as-spun CNTF and (f) CSA-CNTF. Specific modulus-strain curves of (g) as-spun CNTF and (h) CSA-CNTF. The scale bars in (a) and (c) are 5 μm and those in (b) and (d) are 1 μm.

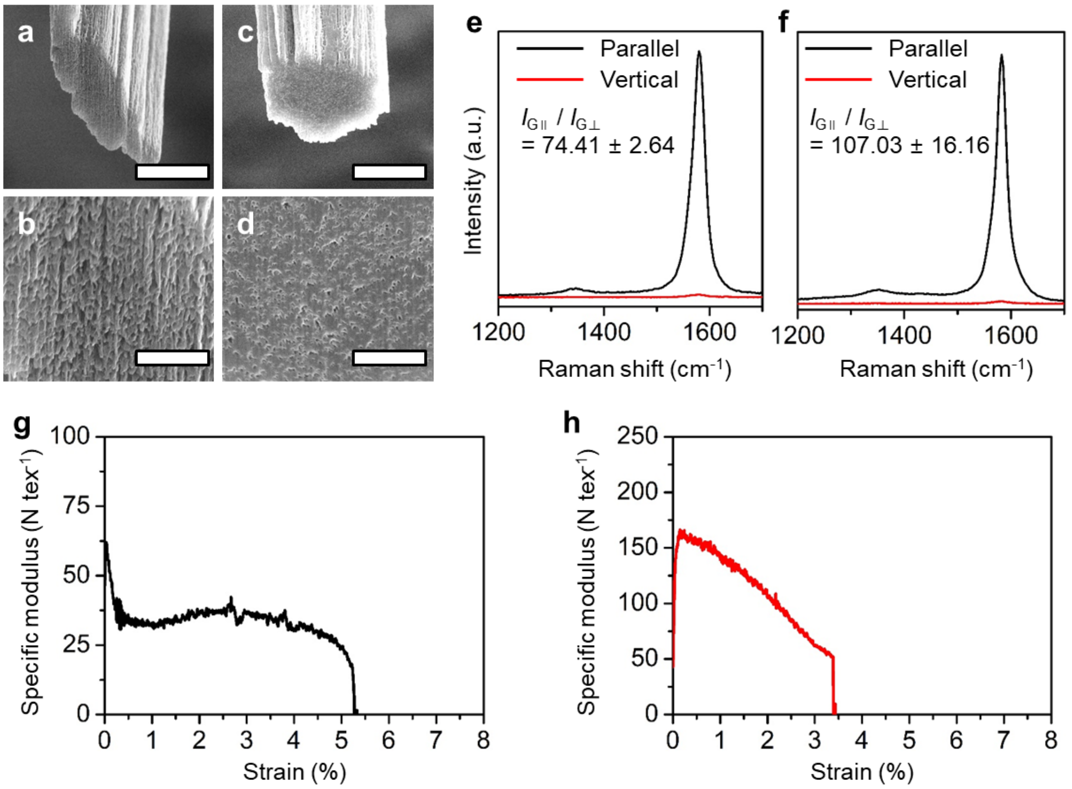

Supplementary Figure 7. Structural change of CNTFs with medium *I*G/*I*D after CSA treatment. Cross-sectional SEM images of (a)-(b) as-spun CNTF and (c)-(d) CSA-CNTF. Polarized Raman spectra of (e) as-spun CNTF and (f) CSA-CNTF. Specific modulus-strain curves of (g) as-spun CNTF and (h) CSA-CNTF. The scale bars in (a) and (c) are 5 μm and those in (b) and (d) are 1 μm.

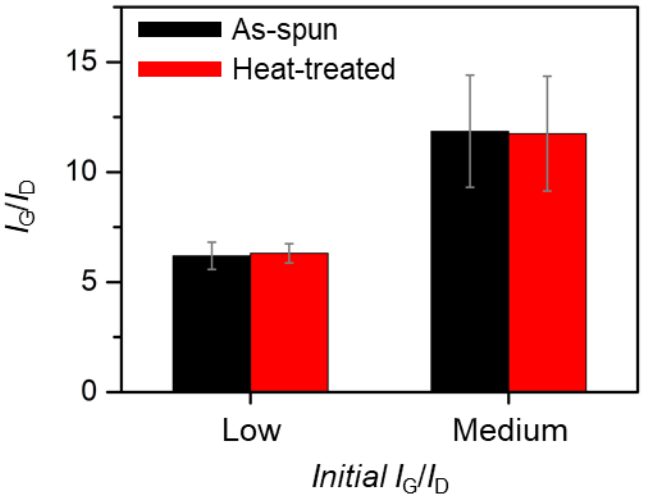

Supplementary Figure 8. Evolution in the defect density after heat treatment. The evolution in the defect density (I_G/I_D) of CNTFs with two levels of initial I_G/I_Ds after heat treatment in air. Error bars represent the standard deviation. Number of replicates is as follows; low and as-spun (n = 5), low and heat-treated (n = 3), medium and as-spun (n = 5), and medium and heat-treated (n = 5).

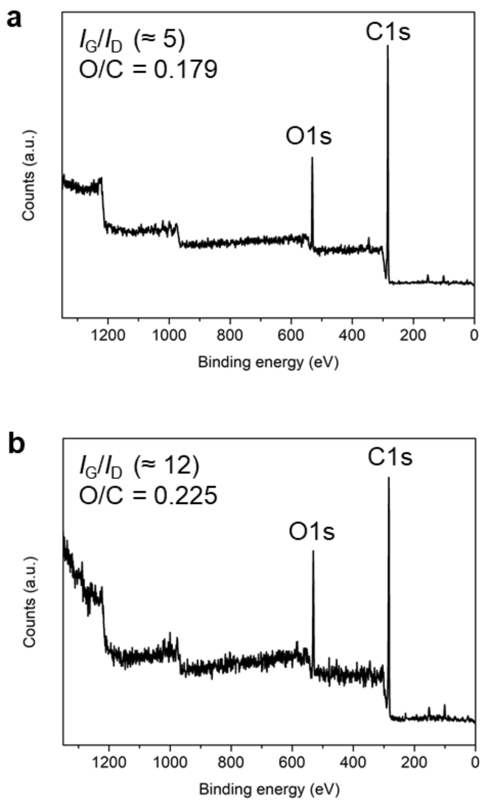

Supplementary Figure 9. Amount of O in CNTFs. X-ray photoelectron spectroscopy (XPS) survey spectra of CNTFs that had (a) low and (b) medium initial I_G/I_Ds after heat treatment in air.

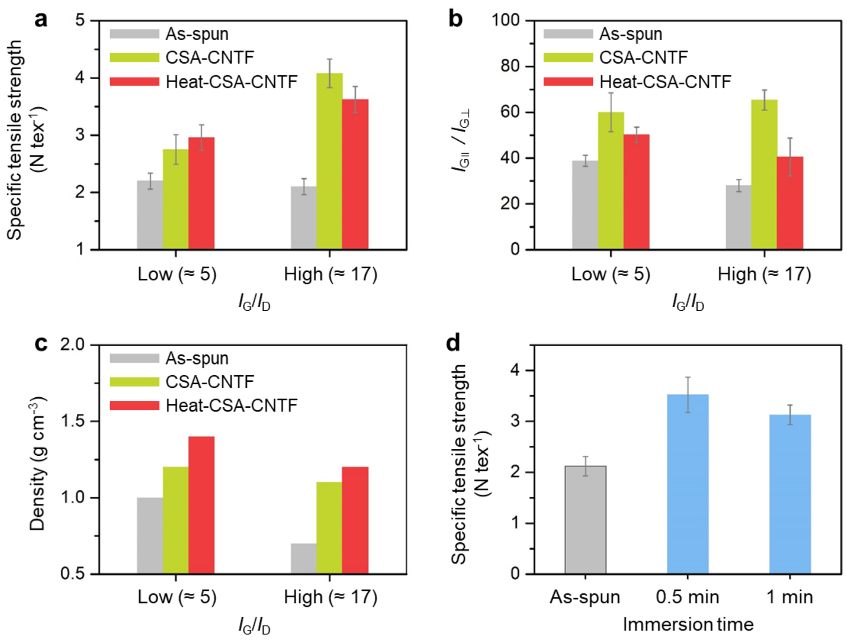

Supplementary Figure 10. Combinational effect of I_G/I_D and heat treatment. (a) Specific tensile strength, (b) $I_{G\parallel}/I_{G\perp}$, and (c) density of as-spun CNTF, CSA-CNTF, and heat-CSA-CNTF with low and high I_G/I_Ds. The immersion time and the stretching ratio in (a)-(c) were 1 min and 10%, respectively. (d) Specific tensile strength of heat-CSA-CNTF that had medium I_G/I_D as a function of immersion time. The stretching ratio in (d) was 10%. These are the real values of the data shown in Fig. 6. Error bars represent the standard deviation. Number of replicates for each experimental condition is as follows; (a) low and as-spun (n = 10), low and CSA-CNTF (n = 9), low and heat-CSA-CNTF (n = 8), high and as-spun (n = 12), high and CSA-CNTF (n =10), and high and heat-CSA-CNTF (n = 11), (b) 5 for all cases, and (d) as-spun (n = 12), 0.5 min (n = 12), and 1 min (n = 6).

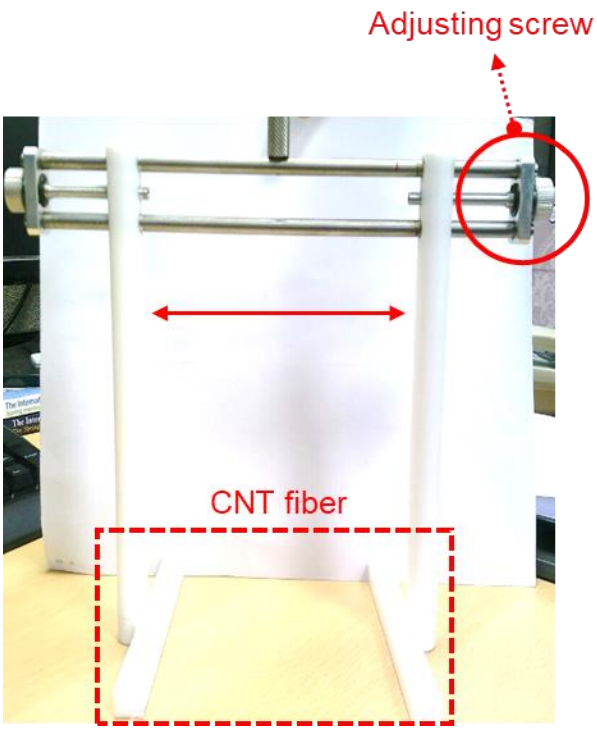

Supplementary Figure 11. The custom-designed apparatus for CSA densification. The custom-designed apparatus for stretching CNTFs in CSA by adjusting the screws.
